# The VASCERN-VASCA Working Group Diagnostic and Management Pathways for Venous Malformations

**DOI:** 10.1097/JOVA.0000000000000064

**Published:** 2023-03-23

**Authors:** Anne Dompmartin, Eulalia Baselga, Laurence M. Boon, Andrea Diociaiuti, Veronika Dvorakova, May El Hachem, Paolo Gasparella, Emir Haxhija, Nader Ghaffarpour, Kristiina Kyrklund, Alan D. Irvine, Friedrich G. Kapp, Jochen Rößler, Päivi Salminen, Caroline van den Bosch, Carine van der Vleuten, Leo Schultze Kool, Miikka Vikkula

**Affiliations:** a Dermatology Department CHU Caen Université Caen Normandie CHU Caen Côte nacre 14033 Caen Cedex, France; b Pediatric Dermatology, Hospital Sant Joan de Deu, Barcelona, Spain; VASCERN VASCA European Reference Centre; c Center for Vascular Anomalies, Division of Plastic Surgery, University Clinics Saint-Luc, University of Louvain, Brussels, Belgium; VASCERN VASCA European Reference Centre; d Dermatology Unit and Genodermatosis Unit, Genetics and Rare Diseases Research Division, Bambino Gesù Children’s Hospital, IRCCS, Piazza Sant’Onofrio 4, 00165, Rome, Italy; VASCERN VASCA European Reference Centre; e Paediatric Dermatology, Children’s Health Ireland; ^y^Clinical Medicine, Trinity College Dublin, Ireland; VASCERN VASCA European Reference Centre; f Department of Paediatric and Adolescent Surgery, Medical University of Graz, Graz, Austria; VASCERN VASCA European Reference Centre; g Department of Plastic and Craniofacial Surgery Karolinska University Hospital Stockholm, Stockholm, Sweden; VASCERN VASCA European Reference Centre; h Department of Pediatric Surgery, Children’s Hospital, University of Helsinki and Helsinki University Hospital, Helsinki, Finland; VASCERN VASCA European Reference Centre; i Division of Pediatric Hematology and Oncology, Department of Pediatrics and Adolescent Medicine, Medical Center - University of Freiburg, Faculty of Medicine, University of Freiburg, 79106 Freiburg, Germany; VASCERN VASCA European Reference Centre; j Division of Pediatric Hematology and Oncology, Department of Pediatrics, Inselspital, Bern University Hospital, University of Bern, Bern, Switzerland; k Department of Pediatric Surgery, HUSRare Disease Center, Helsinki University Hospital and University of Helsinki, Helsinki, Finland; VASCERN VASCA European Reference Centre; l Hevas, Patient Organisation for Vascular Anomalies, Nijkerk, the Netherlands; m Department of Dermatology, Radboudumc Expertise Center for Haemangiomas and Congenital Vascular Malformations Nijmegen (Hecovan), Radboud University Medical Center, Nijmegen, the Netherlands; VASCERN VASCA European Reference Centre; n Department of Radiology, Radboudumc Expertise Center for Haemangiomas and Congenital Vascular Malformations Nijmegen (Hecovan), Radboud University Medical Center, Nijmegen, the Netherlands; VASCERN VASCA European Reference Centre; o Human Molecular Genetics, de Duve Institute, University of Louvain, Brussels, Belgium

**Keywords:** venous malformation, rare disease, vascular anomalies

## Abstract

To elaborate expert consensus patient pathways to guide patients and physicians toward efficient diagnostics and management of patients with venous malformations.

**Methods:**

VASCERN-VASCA (https://vascern.eu/) is a European network of multidisciplinary centers for Vascular Anomalies. The Nominal Group Technique was used to establish the pathways. Two facilitators were identified: one to propose initial discussion points and draw the pathways, and another to chair the discussion. A dermatologist (AD) was chosen as first facilitator due to her specific clinical and research experience. The draft was subsequently discussed within VASCERN-VASCA monthly virtual meetings and annual face-to-face meetings.

**Results:**

The Pathway starts from the clinical suspicion of a venous type malformation (VM) and lists the clinical characteristics to look for to support this suspicion. Strategies for subsequent imaging and histopathology are suggested. These aim to inform on the diagnosis and to separate the patients into 4 subtypes: (1) sporadic single VMs or (2) multifocal, (3) familial, multifocal, and (4) combined and/or syndromic VMs. The management of each type is detailed in subsequent pages of the pathway, which are color coded to identify sections on (1) clinical evaluations, (2) investigations, (3) treatments, and (4) associated genes. Actions relevant to all types are marked in separate boxes, including when imaging is recommended. When definite diagnoses have been reached, the pathway also points toward disease-specific additional investigations and recommendations for follow up. Options for management are discussed for each subtype, including conservative and invasive treatments, as well as novel molecular therapies.

**Conclusion:**

The collaborative efforts of VASCERN-VASCA, a network of the 9 Expert Centers, has led to a consensus Diagnostic and Management Pathways for VMs to assist clinicians and patients. It also emphasizes the role of multidisciplinary expert centers in the management of VM patients. This pathway will become available on the VASCERN website (http://vascern.eu/).

## Introduction

Although classified as “rare diseases,” venous malformations (VMs) are the most frequent vascular malformations seen in specialized multidisciplinary centers for vascular anomalies. Although bluish discoloration and swelling are typical symptoms, the clinical diagnosis may be difficult; some VMs are initially misdiagnosed as a sarcoma or other tumors. Moreover, there are several subtypes of venous anomalies, and genetic bases have been identified for most. As the pathophysiological basis is related to activation of the PI3K-AKT-mTOR pathway, the repurposing of cancer drugs has become possible. This has implications for management options, and these are likely to become subtype-specific depending on the identified mutation.

The Vascular Anomalies working group (VASCA) of the European Reference Network (ERN) for Rare Multisystemic Vascular Diseases (VASCERN) produced a Diagnostic and Management Pathway based on a group of expert interaction to share their experience for the diagnosis and management of these slow-flow vascular lesions.^1,2^ The VASCERN-VASCA diagnostic and management vascular malformation-pathways should help decision-making for physicians suspecting a vascular anomaly. Separate pathways have been developed for severe/rare infantile hemangiomas, lymphatic malformations, and capillary malformations.

## Patients and methods

VASCERN-VASCA is composed of a multidisciplinary panel of experts including dermatologists, pediatric surgeons, pediatric hemato-oncologists, a plastic surgeon, an interventional radiologist, a geneticist, and patient representatives. They represent health care providers endorsed by their national governments (via Ministries of Health) as board members of the ERN for Rare Multisystemic Vascular Diseases (VASCERN) or affiliated members of VASCA research groups. Based on the principle that decisions from a group of experts are better than from single experts, VASCERN-VASCA decided to develop diagnostic and management pathways for VMs using the Nominal Group Technique, a well-established, structured, multistep, facilitated, group meeting technique used to generate consensus.^1,2^

The pathway was developed during 2 face-to-face meetings on October 11–12, 2018, and May 27–28, 2019, with additional online meetings (20th February, 30th March, 17th April, 19th June, 2019) to facilitate discussion. It was further developed by mail in between the online meetings to avoid group dynamics. All VASCA members (the authors) were able to give their remarks and suggestions for revisions directly to the first facilitator. These were discussed during the online meetings. The pathways were finalized on November 15, 2019. Two facilitators were identified: one to propose initial discussion points and draw the pathway draft, and another to chair the discussion (MV). A dermatologist (ADo) was chosen as first facilitator due to her specific particular clinical and research experience. Further decision-points were proposed by the group and best choices were discussed within the panel of experts. Conflicting points were further discussed until consensus was reached by the multidisciplinary team. The chair of the group promoted inputs from all members, summarized the opinions and the reasons for the choices, identifying common ground. No time limit was imposed to reach consensus. A final online VASCA meeting was organized during April 28–29, 2020, to definitely approve the pathways. The pathways were presented as a poster at the International Society for the Study of Vascular Anomalies (ISSVA) 12–15 May 2020 virtual meeting for international expert discussion.

## Results

### Diagnosis of VMs

**Figure 1.**
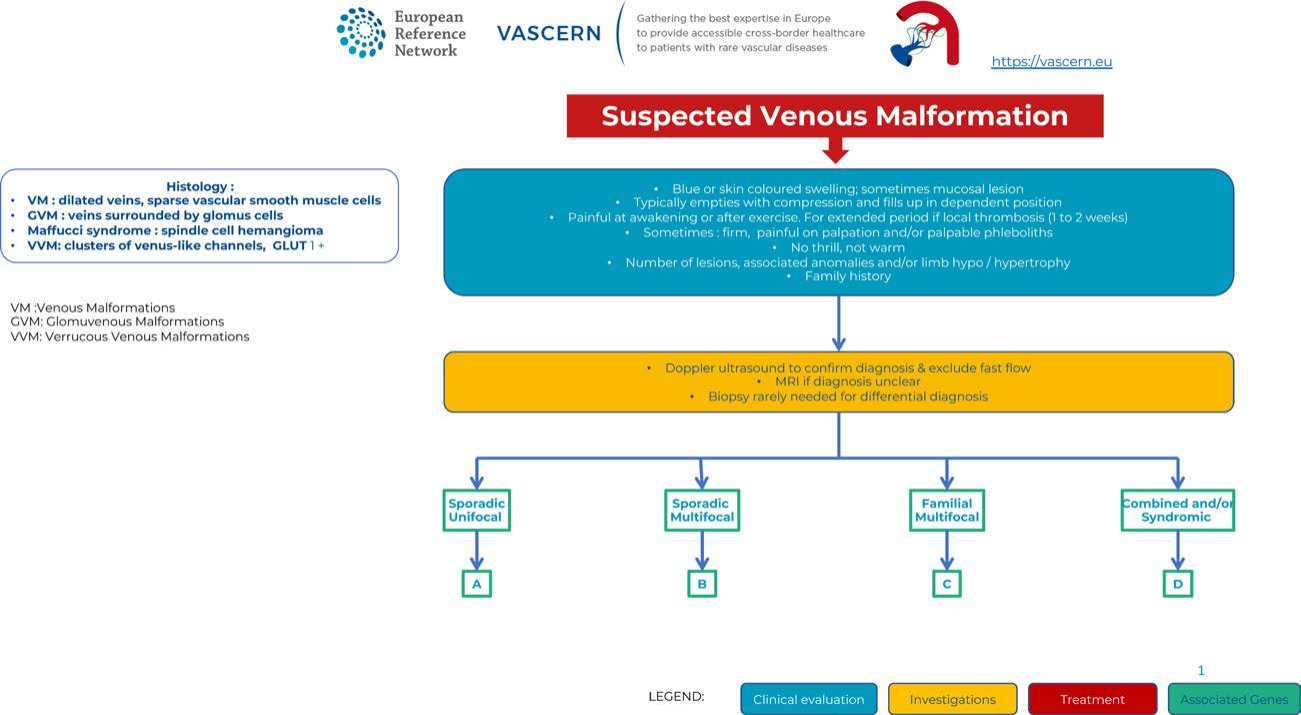
Diagnosis of Venous Malformations.

VMs are light-to-dark-blue lesions that can be emptied by compression or elevation of the affected body part^3–5^ (Figure 1). There is no thrill or murmur. On palpation, the affected area is not warmer than the nonlesional areas. Their consistency is soft and compressible when they are subcutaneous. VMs can affect any tissue or organ, including skin, muscle, joint, intestine, or bone. Depending on their size and location, activity-level and hormonal status of the patient, VMs can be painful. They may also be life-threatening because of consumptive coagulopathy, bleeding, expansion, or obstruction of vital structures.

Palpation is not painful, except in glomuvenous malformation (GVM), unless thrombosis occurs. Most VMs undergo a continuous cycle of spontaneous thrombosis and thrombolysis. VMs do not cause pulmonary embolism when the channels that are thrombosed are not in continuity with the main conducting channels. However, this can occur when large draining veins are present and this may lead to pulmonary hypertension. Persistent thrombi can calcify in the malformation, resulting in phleboliths, pathognomonic for VMs. They can be detected by Doppler ultrasound and magnetic resonance imaging (MRI) examinations confirming diagnosis.

Biopsy is rarely needed but it may be useful to distinguish different slow-flow lesions from vascular and other tumors. Histologically, the different types of VMs (unifocal, multifocal sporadic, or familial) are characterized by enlarged venous channels lined by a single flattened layer of endothelial cells surrounded by sparse, irregularly distributed smooth muscle cells. GVMs, previously known as “glomangiomas,” are characterized by the presence of undifferentiated smooth muscle cells (glomus cells) surrounding convoluted venous channels.^6^ Histologically, Maffucci syndrome vascular lesions are characterized by spindle cells, and thus called “spindle cell hemangiomas.” Verrucous venous malformations (VVMs) are composed of clusters of GLUT1-positive venous-like channels associated with various degrees of epidermal hyperplasia/hyperkeratosis.

### Imaging

Ultrasound is a very useful examination to confirm slow-flow vascularity. In 80% of cases, VMs appear as hypoechoic or heterogeneous and compressible lesions. However, differentiation between venous and lymphatic malformations can be difficult. MRI with spin-echo T1 and fat saturated T2 weighted sequences is the gold standard for pretherapeutic evaluation of VMs. The ultrasound and MRI studies should be extended beyond the clinically visible lesion. MRI-angiography does not add significant information, but may rule out arteriovenous malformations. However, large VMs may also harbor small feeding vessels resulting in a capillary blush that should not be mistaken for an AVM. T1 and T2-weighted with fat saturation MRI images depict the anatomic relation between the vascular lesion and adjacent organs, nerves, tendons, and muscles. D-dimer and fibrinogen tests are useful and highly specific to detect a venous component of a vascular anomaly (pure, combined, or syndromic).^5,7^ Elevated D-dimer levels is present in 50% of patients with sporadic VMs and in almost all multifocal VMs. It helps differentiate multifocal VMs from GVMs and lymphatic malformations (the latter 2 usually having normal D-Dimer levels). This inexpensive biomarker may be useful to follow-up efficacy of treatment. Plain radiographs may be interesting to study bone and joint repercussions (especially at the level of the knee) of a VM and to visualize phleboliths.

### Diagnostic work-up

Once the diagnosis of a slow-flow vascular malformation is suspected, one must search for family history for further classification. Most VMs occur sporadically, are unifocal, and harbor somatic mutations (Figure 1A). Some patients with sporadic VM have multifocal lesions (multifocal sporadic VM), which also harbor somatic mutations (Figure 1B).^8^ Lesions in familial forms, including mucocutaneous venous malformations (VMCMs) and GVMs, are often multifocal and caused by germline mutations with a somatic second-hit (Figure 1C).^8^ Whether sporadic or familial, patients with multifocal lesions can develop additional lesions over time.^9^ VM may also be part of a lesion combined with lymphatic and/or capillary malformation, with or without soft tissue overgrowth (Figure 1D).

### Clinical subtypes

**Figure 2.**
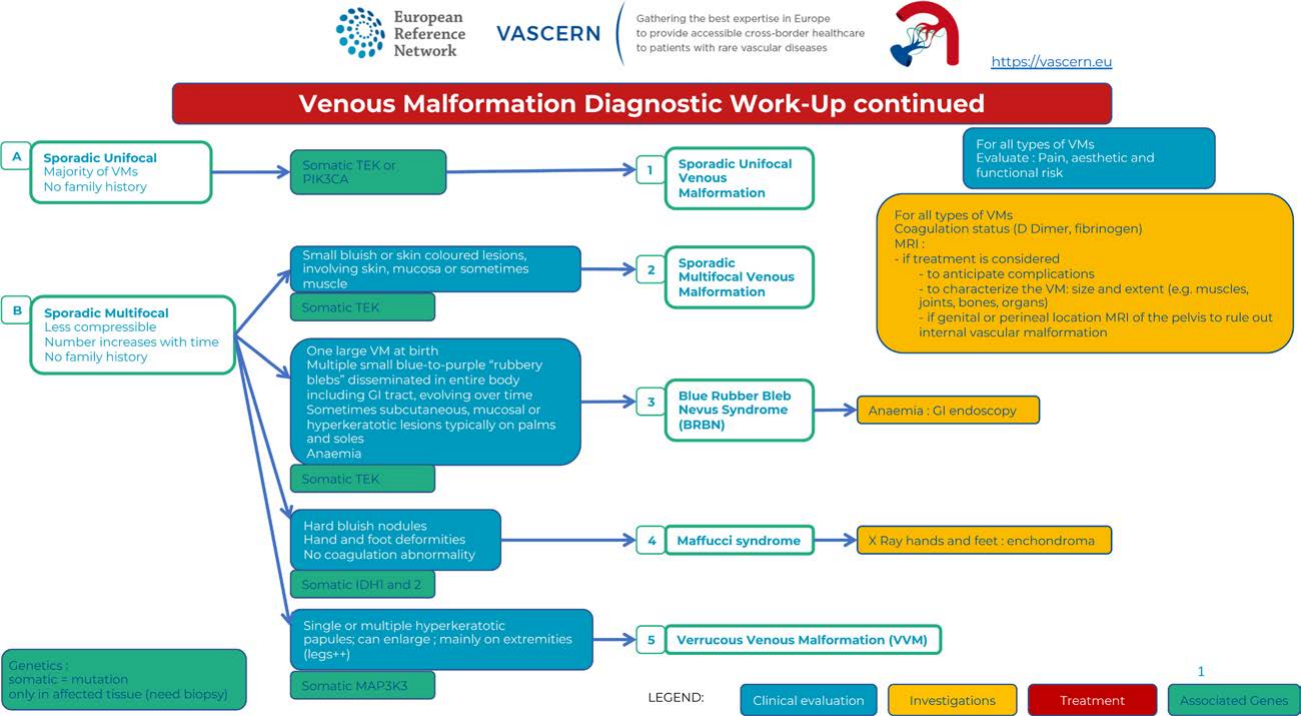
Diagnostic work-up : sporadic lesions.

- Most VMs are sporadic and unifocal, and harbor somatic activating mutations in the *TIE2/TEK* or the *PIK3CA* gene.^8–10^ They can be small or extensive (diffuse) encompassing a whole extremity or even more (Figure 2).

**Figure 3.**
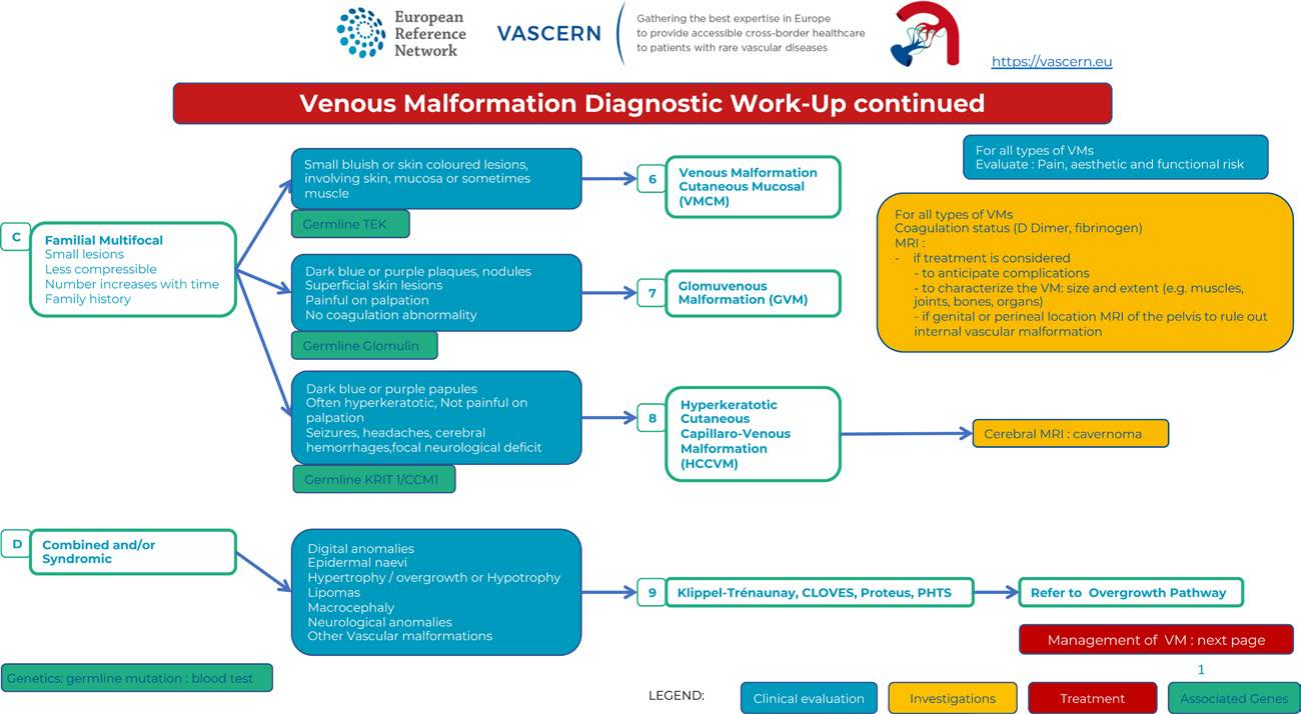
Diagnostic work-up : familial and combined/syndromic lesions.

Sporadic multifocal lesions include multifocal VMs, blue rubber bleb nevus (BRBN), Maffucci syndrome, and VVMs.

- Sporadic multifocal VMs are mostly raised, small in diameter (<5 cm) and less compressible. They harbor a somatic *TIE2/TEK* mutation (Figure 2A).
- The BRBN syndrome (Bean syndrome) associates cutaneous and visceral VMs. The cutaneous VMs are multiple, small, and rubbery, and are frequently located on the palms and soles. A large so called “dominant” lesion is often present at birth, followed by an increasing number of multiple dark blue lesions disseminated all over the body. The multiple lesions of the intestine, which can cause chronic bleeding and anemia, are a diagnostic criterion. In rare cases, BRBN can involve other organs, including the bladder, liver, spleen, kidneys, and lungs.^9^ Vascular lesions have a double (cis) *TIE2/TEK* somatic mutation (Figure 2B3).^11^
- Maffucci syndrome is a rare developmental disorder characterized by multiple enchondromas associated with subcutaneous spindle cell hemangiomas of the distal extremities. The disease becomes symptomatic during childhood with the development of enchondromas of the bones of the hands and feet, as well as of the long bones. Deformities and shortening of the extremities often occur. The subcutaneous vascular nodules appear later, around puberty, on the fingers and the toes; phleboliths may become present. D-dimer levels are normal. Histopathological examination shows features of spindle cell hemangioma, specifically nodules of dense spindle cell infiltration in combination with dysplastic vessels. These patients have a high incidence of malignancies (40%) mainly of chondrosarcoma, but also glioma, fibrosarcoma, and angiosarcoma.^10^ Lesions have *IDH1* or *IDH*2 gene mutations (Figure 2B4).^12^
- VVM is a raised purple hyperkeratotic papule, which can be single or multiple and mainly occur on extremities (91%). Histologically affected tissue is composed of venous-like channels with a positive GLUT1 immunostaining. They often have a somatic *MAP3K3* mutation (Figure 2B5).^13^

Familial forms are caused by germline mutations associated with a somatic second-hit and are often multifocal; they comprise VMCMs (<1% of all VMs), GVMs (5% of all VMs), and hyperkeratotic cutaneous capillary-venous malformations (HCCVMs <1% of all VMs). VMCMs are caused by mutations in *TIE2/TEK* gene,^14^ GVMs by mutations in the Glomulin gene (*GLMN*)^15^ and HCCVMs by mutations in the *KRIT1/CCM1* gene^16^ (Figure 3).

- In contrast to sporadic VMs, VMCMs are more superficial and rarely invade muscles. The extension within a joint or a bone has not been reported. Due to their small size, they are often asymptomatic.^14^ Most of them have elevated D-dimer levels (Figure 3C6).
- GVMs differ clinically and genetically from VMs.^15^ Their color varies from pink to purple or dark blue. They are present at birth, can expand in size and number throughout life and are raised, cobblestone or plaque-like and slightly hyperkeratotic. In contrast to VMCMs, they are mainly located in the extremities and not easily compressible. They are more superficial than VMs, involving both the skin and subcutis, and rarely mucosal. GVMs are painful when compressed and thus, contrary to VMs, elastic compressive garments aggravate pain. They should not be confused with the solitary and painful glomangioma of the nail bed. In contrast to VMCMs, they have normal D-dimer levels and a *GLMN* (glomulin) germline mutation (Figure 3C7).
- HCCVMs have been considered specific to the familial cavernous cerebral malformations syndrome. They are difficult to distinguish clinically from VVM as they are plaque like, irregularly shaped, purple or black and mainly located on the limbs. They are associated with germline *KRIT1/CCM1* mutations (Figure 3C8).^16^

### Management

**Figure 4.**
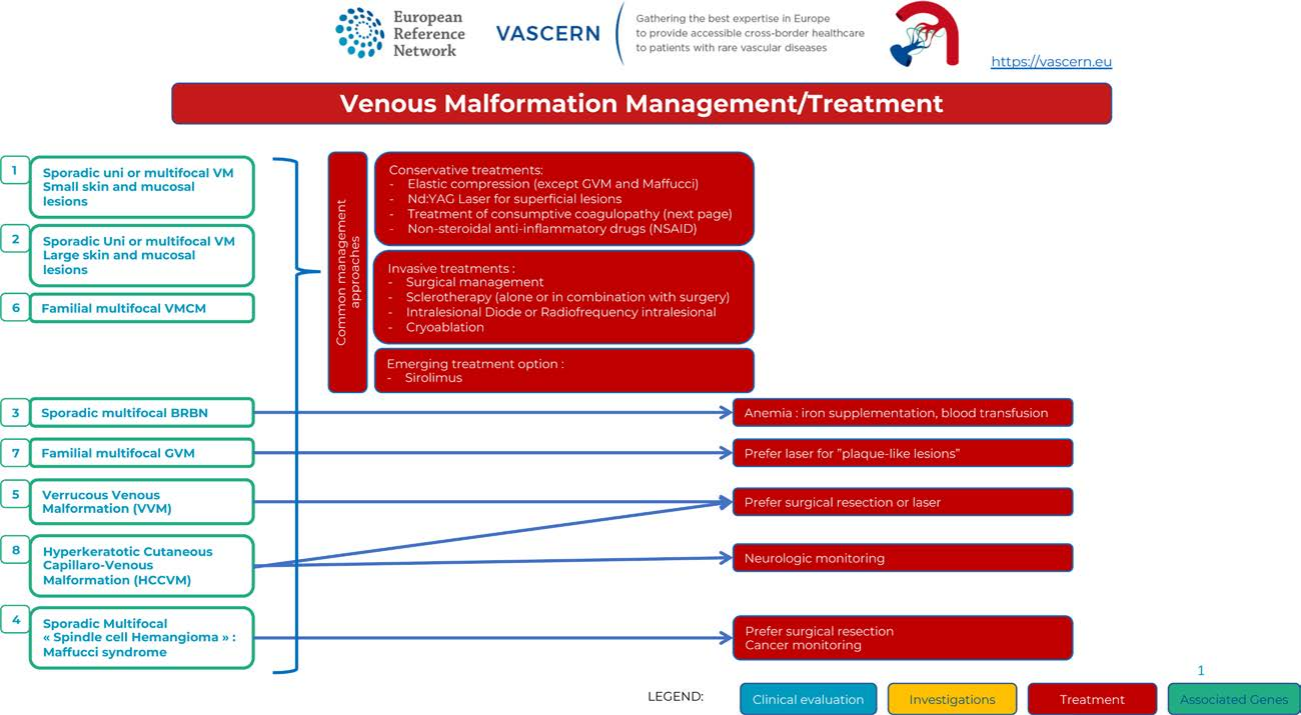
Management/treatment of Venous Malformations.

Management of VMs needs interdisciplinary discussion and there is no available “standard” treatment algorithm and combinations of different treatment modalities is often offered. The common management approach is targeted toward the clinical symptoms at different times of evolution. Tailored compression-garment is the first-line treatment for symptomatic and extensive VMs of the extremities to reduce pain and thrombosis. It is contraindicated in GVMs, as it increases pain. It is also not effective in Maffucci syndrome. Nonsteroidal anti-inflammatory drugs are proposed if pain is not relieved, or if compression is not anatomically possible (Figure 4).

Curative treatment is rarely possible; yet invasive treatments are often useful: Surgical excision is proposed in the following situations: small VMs or GVMs with possible complete surgical excision, VMs with well-defined margins independent on their size but with the goal of complete resection without postoperative functional deficits, or VMs with postsclerotherapy fibrosis. Partial resection without preceding sclerotherapy is rarely performed because of the risk of relapse and/or surgical morbidity. Surgery is the treatment of choice for GVMs because they are more superficial with a limited invasion to the adjacent structures, although sclerotherapy may decrease pain. Percutaneous sclerotherapy is the gold standard treatment to reduce the volume of VMs. The goal is to obliterate the channels by causing damage to the endothelium with subsequent inflammation and fibrosis. Intralesional diode or radiofrequency and cryoablation are newer techniques with promising results. Neodymium-doped Yttrium Aluminium Garnet Laser is also potentially useful for superficial lesions (small superficial VMs, superficial discoloration of larger VMs, some GVMs and HCCVMs).

### Management of chronic consumptive coagulopathy

**Figure 5.**
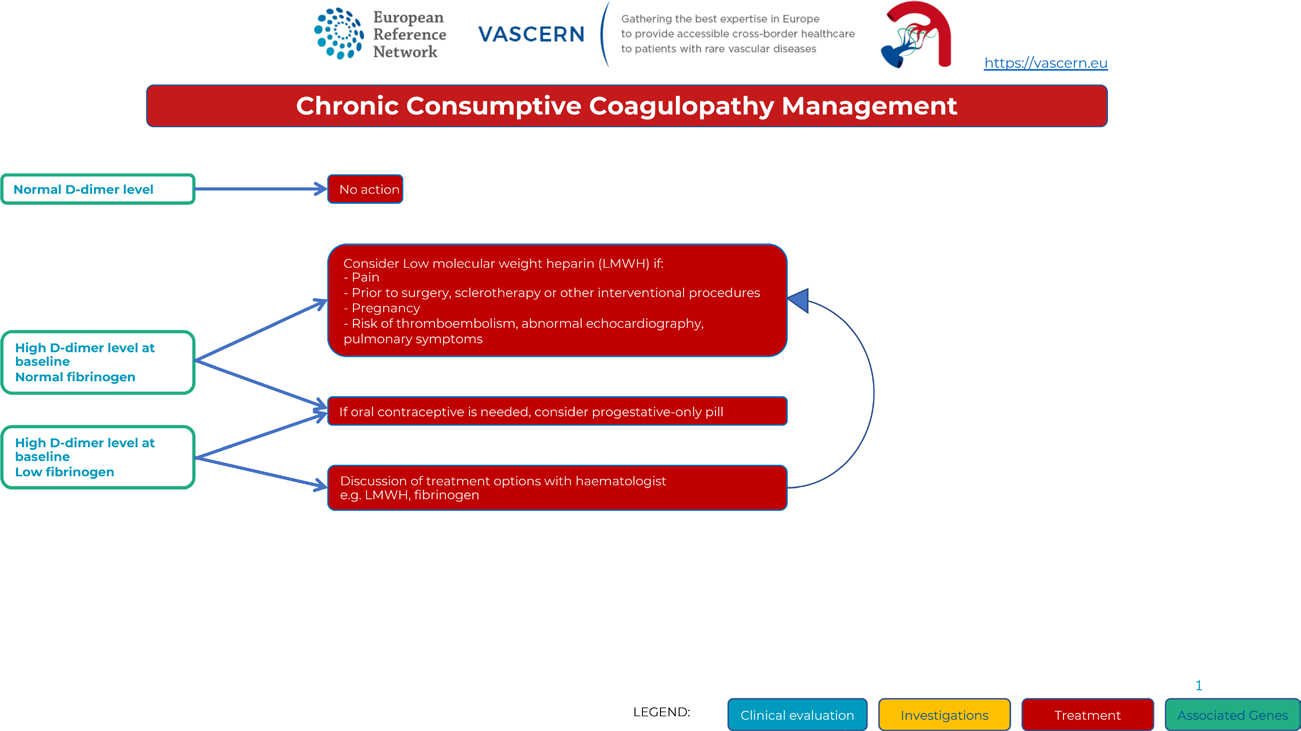
Chronic Consumptive Coagulopathy Management in Venous Malformations.

VMs can be associated with dramatically increased D-dimer levels in otherwise healthy patients due to chronic consumptive coagulopathy (also called as localized intravascular coagulopathy). When fibrinogen level is concomitantly low, fibrinolysis is even more elevated with an increased risk for bleeding. Such coagulopathy is usually well tolerated in everyday life, but systemic activation of coagulation can occur during surgical resection or other interventional treatments and may progress to disseminated intravascular coagulopathy. Each episode of intralesional thrombosis may lead to painful enlargement of a VM. There is no available treatment algorithm for the management of chronic consumptive coagulopathy. Assessment of coagulation status is needed for all patients with VMs. If D-dimer levels are normal, no treatment is required. If D-dimer levels are elevated and associated with normal fibrinogen, low molecular weight heparin can be proposed when VM is painful or when the thrombotic risk is increased: pregnancy, planned surgery, sclerotherapy or interventional procedure, abnormal cardiac echography (elevated pulmonary arterial pressure), and/or pulmonary symptoms. If fibrinogen is low, discussion of low molecular weight heparin treatment with hematologists is necessary. Direct oral anticoagulants are currently being explored as a possible curative or preventive treatment for intravascular coagulopathy associated with slow flow vascular malformations.^17^ As oral contraceptives may increase thrombosis and chronic consumptive coagulopathy, a progesterone-only pill may be favored (Figure 5).

### Genetic and molecular mechanisms

Disturbances in the PI3K/AKT/mTOR pathway are associated with VMs, 60% of them being caused by gain-of-function somatic mutations in the *TEK* gene (which encodes Tie2 receptor) and 20% in the *PIK3CA* gene. Elucidation of underlying genetic and molecular mechanisms has led to better pathophysiological understanding of these lesions and the use of targeted treatments. Medical treatment will likely be an effective addition (or alternative) to the invasive treatments for these often difficult-to-treat entities. The mTOR inhibitor sirolimus is the most extensively studied drug in this context so far^18^ and should be considered in patients with extensive disease, gastrointestinal bleeding in BRBN and VMs refractory to other treatment modalities. New drug therapies that are being repurposed from oncological treatments (such as PIK3CA inhibitors, including Alpelisib) are also being studied.

## Discussion

In the absence of clinical trial and meta-analysis in the field of rare conditions, expert opinion is the best tool to generate algorithms to improve the quality of diagnostic procedures and management of patients. Indeed, in rare diseases level V evidence is still a necessary means to answer to a clinical question.

The Nominal Group Technique is a consensus technique that involves a group of experts to generate ideas and determine priorities.^1,2^ It is “a structured meeting which seeks to provide an orderly procedure for obtaining qualitative information from target groups who are most closely associated with a problem area.” The quality of the statements by an expert panel depends on the members’ skills. Using the VASCERN network, the group expertise in the field of vascular anomalies was guaranteed by the selection of national reference centers endorsed by their governments and selected by the European Community’s ERN network on the basis of well-defined criteria. These healthcare providers have representatives who are members of the ISSVA.

In addition to these pathways, we can pinpoint that

- intramuscular VM is often misdiagnosed as a muscular strain after exercise induced pain
- VMs may be misdiagnosed as a tumor (sarcoma is often suspected)
- one should avoid unnecessary complementary exams (those not mentioned in the pathways)
- if imaging is performed before treatment (eg, to search for associated malformations or to evaluate infiltration of neighboring vital structures, such as pelvis extension of a perineal VM) one should use at least T1/T2 fat sat MRI, when it is necessary to define the tissues involved and the extent of the VM
- one should avoid arteriography, as arteries are normal
- blood tests should include coagulation status with D-dimer and fibrinogen levels
- precise diagnosis of the type of slow-flow vascular malformation is needed for correct management
- asymptomatic VMs do not need to be treated

In conclusion, the VASCERN-VASCA proposes an expert opinion on diagnostic and management pathways of VMs as useful tools to improve the diagnosis and management of these patients. It focuses on the importance of the careful clinical examination and the need of appropriate investigations to confirm the diagnosis. The proposed management is currently used in multidisciplinary centers and will be updated according to new discoveries, especially in the field of genetics, which is leading to development of theragnostic approaches in the management of slow-flow anomalies.^8^

## Acknowledgments

The authors of this publication are members of the Vascular Anomalies Working Group (VASCA WG) of the European Reference Network for Rare Multisystemic Vascular Diseases (VASCERN) - Project ID: 769036. We acknowledge all the patient representatives, who are active members of VASCERN and have contributed significantly to the algorithm. The members of the VASCERN VASCA are as follows: Eulalia Baselga, Laurence M. Boon, Andrea Diociaiuti, Veronika Dvorakova, May El Hachem, Paolo Gasparella, Emir Haxhija, Nader Ghaffarpour, Kristiina Kyrklund, Alan D. Irvine, Friedrich G. Kapp, Jochen Rößler, Päivi Salminen, Caroline van den Bosch, Carine van der Vleuten, Leo Schultze Kool, Miikka Vikkula.
